# Identification of *Streptococcus parasanguinis* DNA contamination in human buccal DNA samples

**DOI:** 10.1186/1756-0500-6-481

**Published:** 2013-11-22

**Authors:** Istiak Mahfuz, Wei Cheng, Stefan J White

**Affiliations:** 1 Centre for Genetic Diseases, Monash Institute of Medical Research, Monash University, 27-31 Wright Street, Clayton 3168, VIC, Australia; 2 Department of Paediatrics, Southern Medical School, Faculty of Medicine, Nursing and Health Sciences, Monash University, Melbourne, Australia; 3 Department of Surgery, Southern Medical School, Faculty of Medicine, Nursing and Health Sciences, Monash University, Melbourne, Australia; 4 Department of Surgery, Beijing United Family Hospital, Beijing, China

## Abstract

**Background:**

The use of buccal swabs in clinical and scientific studies is a very popular method of collecting DNA, due to its non-invasive nature of collection. However, contamination of the DNA sample may interfere with analysis.

**Findings:**

Here we report the finding of *Streptococcus parasanguinis* bacterial DNA contamination in human buccal DNA samples, which led to preferential amplification of bacterial sequence with PCR primers designed against human sequence.

**Conclusion:**

Contamination of buccal-derived DNA with bacterial DNA can be significant, and may influence downstream genetic analysis. One needs to be aware of possible bacterial contamination when interpreting abnormal findings following PCR amplification of buccal swab DNA samples.

## Findings

Buccal swabs are a popular, inexpensive and non-invasive method of collecting DNA samples. It is a convenient procedure for collecting DNA from geographically isolated populations for larger cohort studies [1], and has the advantage of avoiding the stressful process of venepuncture. When using buccal swab DNA, sampling or processing considerations may be important for obtaining optimal results [2]. If buccal swabs are not collected and/or handled properly, potential complications may occur during subsequent analysis [3]. Problems that can affect the interpretation include contamination, degradation, and insufficient yield [4-6]. Here we report a significant *Streptococcus parasanguinis* DNA contamination in human buccal-derived DNA. This bacteria is the most abundant microorganism in the mouth, and a primary colonizer of human tooth surfaces [7,8] that plays an important role in dental plaque formation [9,10].

**Figure 1:**
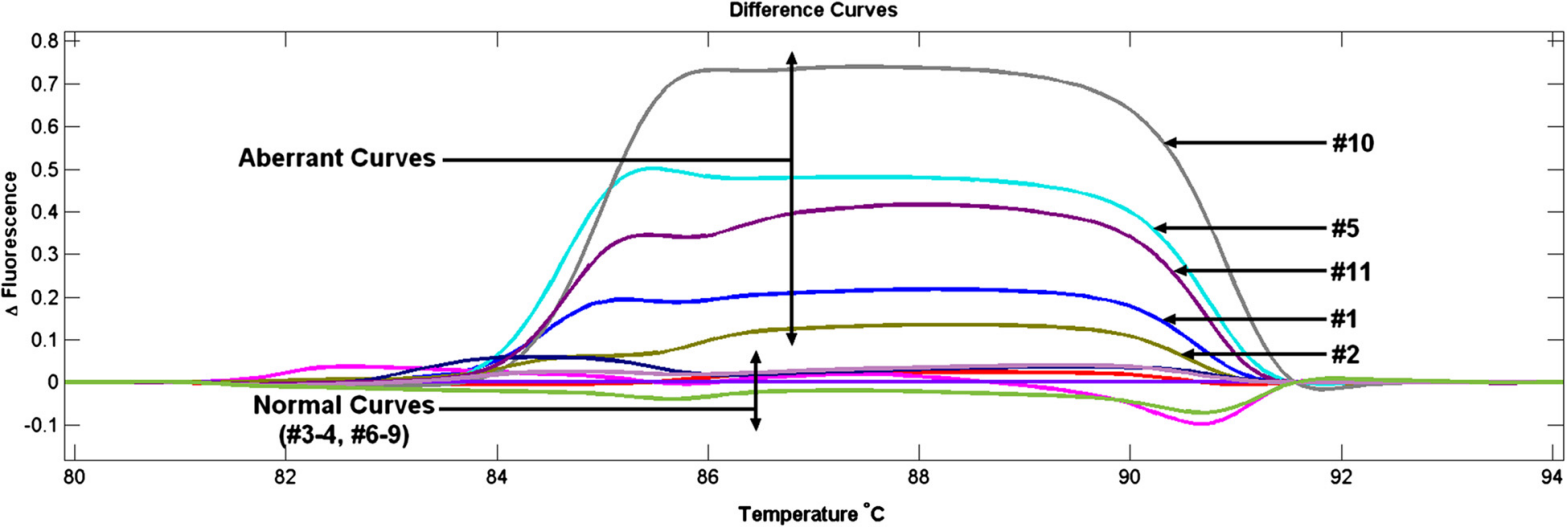
**High Resolution Melt (HRM) analysis of PCR products from human buccal-derived DNA samples.** The aberrant HRM curves are due to contamination with *Streptococcus parasanguinis* DNA.

**Figure 2:**
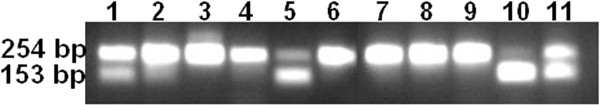
**Gel electrophoresis of the PCR products shown in Figure **1**.** The expected product from the human DNA is 254 bp, with the 153 bp product matching *Streptococcus parasanguinis* DNA.

**Figure 3:**
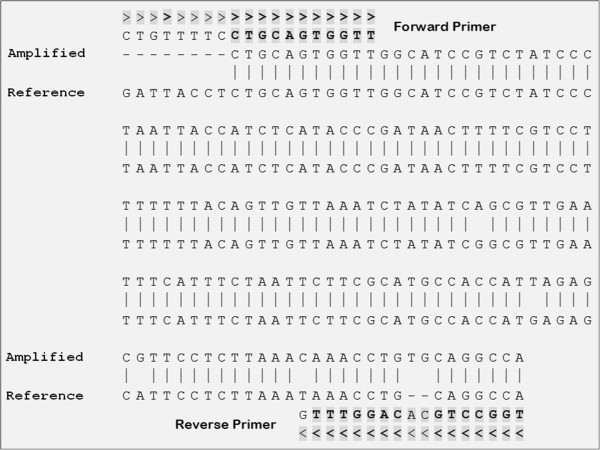
**Alignment of the 153 bp PCR product with the *Streptococcus parasanguinis *plasmid pFW213 reference sequence.** The sequences corresponding to the PCR primers are indicated in bold.

Contamination was detected during mutation screening of the Desmoplakin (*DSP*) gene in buccal DNA of Bladder Exstrophy Epispadias Complex (BEEC) patients. Use of samples for research was approved by the Research Ethics Committee of Royal Children’s Hospital (Approval Number# HREC28140A), Melbourne, Australia. Informed consent was also obtained from the patients or parents/guardians. The intention was to screen *DSP* exon 4 for potential sequence variants using High Resolution Melting (HRM) and Sanger Sequencing. For this, we designed exon-specific forward (5′ CTGTTTTCCTGCAGTGGTT 3′) and reverse (5′ TGGCCTGCACAGGTTTG 3′) primers, predicted to generate a 254 bp product. HRM was performed on 22 samples as previously described [11]. Five samples gave aberrant curves with HRM (Figure 1), and agarose gel analysis showed the presence of two bands in matching samples (Figure 2). Sanger sequence analysis showed that the 153 bp fragment did not align with any human sequence. Alignment with other organisms showed 98% identity with *Streptococcus parasanguinis* plasmid pFW213 [GenBank:EU685104.1] (Figure 3).

To our knowledge, there have been no previous reports of *Streptococcus parasanguinis* contamination affecting genetic analysis of buccal swab DNA. It is not unexpected to have Streptococcal DNA contamination in DNA from a human buccal swab, due to the high abundance in oral microflora [12,13]. In addition, when a buccal sample is collected, researchers generally do not enquire whether there is any oral disease. This might influence the quality of DNA isolated from buccal swabs. Under such circumstances, a blood sample may be a preferable method for obtaining DNA. A comparison of blood- and buccal-derived DNA from a Danish nurse cohort has previously been reported. 100% of the blood-derived DNA samples could be genotyped or PCR amplified, whereas only 23% of the DNA samples from mouth swabs could be PCR amplified and none of the swab-derived DNA samples could be genotyped [14].

Our finding demonstrates that bacterial contamination may be a significant contributor to the total amount of DNA isolated from a buccal swab, and may explain why an apparently sufficient quantity of high quality DNA does not perform as expected in human-specific PCR amplifications.

## Competing interests

The authors declare that they have no competing interests.

## Authors’ contributions

IM performed the molecular analysis and drafted the manuscript. WC helped in coordination and drafting of the manuscript. SW conceived of the study, participated in its design and coordination and drafted the manuscript. All authors read and approved the final manuscript.

## Authors’ information

Wei Cheng and Stefan J White: Joint senior authors.
